# Widespread nasal carriage of *Mycobacterium leprae* among a healthy population in a hyperendemic region of northeastern Brazil

**DOI:** 10.1590/0074-02760150178

**Published:** 2015-11

**Authors:** Luana Nepomuceno Gondim Costa Lima, Cristiane Cunha Frota, Rosa Maria Salani Mota, Rosa Livia Freitas Almeida, Maria Araci de Andrade Pontes, Heitor de Sá Gonçalves, Laura Cunha Rodrigues, Carl Kendall, Ligia Kerr

**Affiliations:** 1 Instituto Evandro Chagas, Seção de Bacteriologia e Micologia, Belém, PA, Brasil; 2 Universidade Federal do Ceará, Faculdade de Medicina, Departamento de Patologia e Medicina Legal, Fortaleza, CE, Brasil; 3 Universidade Federal do Ceará, Departamento de Estatística e Matemática Aplicada, Fortaleza, CE, Brasil; 4 Universidade Federal do Ceará, Faculdade de Medicina, Departamento de Saúde Comunitária, Fortaleza, CE, Brasil; 5 Centro de Referência Nacional em Dermatologia Sanitária Dona Libânia, Fortaleza, CE, Brasil; 6 London School of Hygiene & Tropical Medicine, Faculty of Epidemiology and Population Health, London, UK; 7 Tulane University, School of Public Health and Tropical Medicine, Department of Global Community Health and Behavioral Sciences, New Orleans, LA, USA

**Keywords:** leprosy, *Mycobacterium leprae*, nasal carriage, case-control, RLEP PCR, Brazil

## Abstract

A case-control study was conducted to determine the presence of *Mycobacterium leprae* DNA in nasal secretions of leprosy cases and nonleprosy individuals in Fortaleza, Brazil. It included 185 cases identified by physicians at the Dona Libânia National Reference Centre for Sanitary Dermatology (CDERM). A control group (Co) (n = 136) was identified among individuals from CDERM not diagnosed as leprosy cases. To augment the spatial analysis of M. leprae specific repetitive element (RLEP) positive prevalence, an external group (EG) (n = 121), a convenience sample of healthy students, were included. Polymerase chain reaction for the RLEP sequence was conducted for all participants. Prevalence of RLEP positivity for cases and Co were 69.2% and 66.9%, respectively, significantly higher than for EG (28.1%), and reported elsewhere. Male sex, belonging to a lower socioeconomic status (D/E), history of a previous contact with a case and being older, were associated with being a leprosy case. Our geographical analysis demonstrated that the bacillus is widespread among the healthy population, with clusters of RLEP positive multibacillary cases concentrated in distinct areas of the city. Our results suggest that in endemic areas, as in Fortaleza, surveillance for both nonhousehold leprosy contacts and members of the general population living in cluster areas should be implemented.

In the last 20 years, programs for global leprosy control made real progress, with over 14 million people cured of the disease through multidrug therapy (MDT). Although this correlates with a reduction of over 90% in the prevalence rate, transmission still occurs with about 250,000 new cases of leprosy still being reported annually, concentrated in a few countries (WHO 2014). Clusters of high endemicity still remain in Angola, Brazil, the Central African Republic, India, Madagascar, Nepal and the United Republic of Tanzania and in previously highly endemic countries, such as the Democratic Republic of the Congo and Mozambique.

Brazil reported a 65% reduction in the prevalence from 2002-2012, from 4.33 in 2002 to 1.51 cases/10,000 in 2012. Despite this achievement, a few regions in Brazil are still considered endemic, including the North, Northeast and Central-West. A second issue is the reported number of cases in children less than 15 years of age. Of total cases from 2012, 2,246 (7%) of them were younger than 15, resulting in a high detection rate of 17.2/10,000 in this population (tabnet.datasus.gov.br/cgi/deftohtm.exeidb2012/d0206.def).

The state of Ceará (CE) is located in northeastern Brazil. In 2013, 87.5% (161/184) of municipalities in CE diagnosed new cases of leprosy, 20.6% had more than 10 new cases and 12.5% are classified as hyperendemic and recorded over 40 new cases per 100,000 inhabitants. Fortaleza, the state capital, reported 689 cases in the same year, which represents an incidence rate of 27.2 cases/100,000 inhabitants. Even though ≥ 80% of household contacts of cases should be examined as part of leprosy control activities, only 52.3% of contacts were examined in Fortaleza in 2013. Since 5.9% of the total cases reported annually in the state still occur among those less than 15 years of age or younger one could argue that active leprosy transmission is ongoing in CE (SESA 2014).

*Mycobacterium leprae* identification is difficult due to the inability of the bacillus to grow in vitro, thus leprosy diagnosis is based on microscopic detection of the bacilli combined with clinical assessment. DNA studies using polymerase chain reaction (PCR) have been used for the molecular diagnosis of *M. leprae*(Santos et al. 1999, Job et al. 2008). The literature describes several genomic target sequences specific for *M. leprae* that have been used in PCR or as DNA probes*, *including the genes encoding the 36-kDa antigen, 18-kDa antigen, 16S rRNA and the repetitive element sequences of *M. leprae*(Parkash et al. 2004, Martinez et al. 2009, Davis et al. 2013).

PCR has shown high sensitivity and specificity. It is able to detect 100% of multibacillary (MB) and 34-80% paucibacillary (PB) cases in nasal secretions (Scollard et al. 2006). It is also able to detect nucleic acid of *M. leprae* in 5-12% and 1-2% of the contacts of MB and PB cases, respectively (Banerjee et al. 2010). Contacts of a leprosy case from endemic regions are putative carriers of *M. leprae* bacilli. Surveillance of the contacts and known cases can identify new cases, lead to treatment and prevent new cases.

This study has two aims: first, to document the presence of *M. leprae*DNA in nasal secretion of leprosy cases and nonleprosy individuals and, second, to discuss the role of geographic location in the epidemiology of leprosy in Fortaleza.

## SUBJECTS, MATERIALS AND METHODS

*Settings* - Fortaleza is divided into six administrative regions (I-VI). Region II presents the highest mean family income in Fortaleza (average monthly income = US$ 911 in mid-2012 adjusted dollars) and the lowest leprosy incidence. Region V (average monthly income = US$ 232) presents the lowest mean family income and highest leprosy incidence (IPECE 2014). A cross-sectional study was conducted from June 2009-December 2010.

*Subjects* - During the period of the study, 837 new leprosy cases living in Fortaleza were diagnosed by trained dermatologists of the Dona Libânia National Reference Centre for Sanitary Dermatology (CDERM). Since recruitment was conducted two days per week (December, January, July and holidays had reduced recruitment), 185 leprosy cases, identified as C, were included and confirmed by clinical skin examination, skin smear and biopsy. They were classified using Ridley-Jopling criteria (Ridley & Jopling 1966) based on histology and bacterial indexes (BI). Controls (Co) (n = 136) were patients attending in CDERM for other clinical dermatological conditions such as psoriasis, skin cancer or aesthetic blemishes and were not diagnosed with leprosy. Nasal samples were collected for all participants of the study.

In order to explore the role of geographic location and social class in the epidemiology of leprosy in Fortaleza and in response to the high overall rate and potential for laboratory contamination of samples producing this high rate, we included an external group (EG) (n = 121) of medical students in the 1st year class of a private medical school, who reported no history of a previous contact with a leprosy case. Their samples were collected at the same time of the year using the same methodology applied to other participants. We compared this group - living in a geographically separate, high socioeconomic status (SES) area of Fortaleza (region II) - with the C and Co participants who live in poorer areas of the city (regions V and I). Average income in region II is 15.3 times higher than in region V (IPECE 2014). Leprosy prevalence in region V is more than four times higher than in region II (1.4 vs. 0.3 cases/10,000 inhabitants) in 2014 (Geluk et al. 2012). Throughout this article the EG is treated as a separate population, i.e., not a Co group. C and Co participants completed a questionnaire to collect demographic, socioeconomic (ABEP 2014), environmental and behavioural data.

*Laboratory methods* - Nasal samples were obtained from all participants by gently rubbing a nasal swab previously wetted with Tris-EDTA buffer (pH 8.0) in the vestibule on each side of the nose. After collection, each swab was immersed in a sterile and labelled tube and stored at -20ºC until processing. Briefly, each swab was directly cut and collected in a previous labelled tube with 1 mL of 4% NaOH and the remaining Tris-ethylenediamine tetraacetic acid (EDTA) buffer. After centrifugation for 20 min at 2,500 rpm, 0.6 mL of lysis buffer (10 mM Tris-HCl, 10 mM EDTA, 50 mM NaCl, 2% w/v sodium dodecyl sulfate and 0.3 mg/mL proteinase K) was added and the tube was left under incubation for 18 h at 56ºC. Phenol-chloroform-isoamyl alcohol (0.6 mL, 25:24:1) was added and the tube was homogenised by inversion. After centrifugation for 5 min, the aqueous phase was collected and mixed with an equal volume of chloroform. After another brief centrifugation, the upper phase was collected and the DNA was then precipitated with 2.5 mM NaCl and isopropanol. The precipitated DNA was finally eluted in 70 µL of dH_2_O.

To detect the *M. leprae* DNA, three primers were devised, which comprised outer and inner nested pairs based on the *M. leprae*-specific repetitive element (RLEP)2 (X17152). The primers RLEP1 (5'-ATATCGATGCAGGCGTGAG-3') and RLEP2 (5'-GGATCATCGATGCACTGTTC-3') were used to amplify a 282-bp fragment and the inner primer RLEP3 (5'-GGGTAGGGGCGTTTTAGTGT-3') and outer primer RLEP2 to amplify a 238-bp fragment. In order to confirm the results, a separate PCR reaction was conducted using a set of primers targeting the RNA polymerase sigma factor (*rpoT - *Q59532) of the *M. leprae* DNA. It generated a fragment of 91-bp (Matsuoka et al. 2000) (forward: 5'-ATGCCGAACCGGACCTCGACGTTGA-3' and reverse: 5'-TCGTCTTCGAGGTCGTCGAGA-3'). Significantly, both primer sets presented the same melting temperature. Therefore, the PCR amplification conditions for both fragments were the same as described below.

Illustra PuRe Taq Ready-To-Go^TM^ PCR Beads pre-dispensed in 0.25 mL tubes (GE Healthcare) were used in the PCR reaction. Reaction mixtures (25 µL) were prepared by adding 50 nM of each primer (Life Technologies^TM^) and 5 µL of the purified DNA from nasal samples to each tube containing a PCR bead. For the second reaction, 0.5 µL of the PCR product was used as a template. The mixture for both reactions was cycled through the following temperature profile: 94ºC for 5 min followed by 40 cycles at 94ºC for 30 s, 59.5ºC for 30 s and 72ºC for 1 min. The reaction mixture was held at 4ºC before electrophoresis of the products. In each run, a positive control of 20 pg of chromosomal *M. leprae *DNA was included, as was a negative control without target DNA. All incubations were performed in the same thermal cycler.

After amplification was finished, the reaction mixture was run in a 2% agarose gel. After electrophoresis, the gel was stained with ethidium bromide solution and the fragments were examined under the ultraviolet illumination. The PCR products were purified using the QIAquick PCR Purification Kit prior to sequencing in an Applied Biosystems DNA sequencer (Perkin-Elmer Applied Biosystems) using a BigDye Terminator Cycle Sequencing kit. The sequences were identified using SecScape software v.2.7 (Applied Biosystems). A reference RLEP2 sequence (GenBank accession NC002677) was used to align the sequences.

All procedures were conducted by the same technician and used the same methods. False-positive amplifications were addressed by using individual sterile section-cutting blades for swab cutting and sterile glassware for each swab sample. Physical separation of the areas for the handling of samples, PCR preparation and PCR analysis was assiduously maintained throughout the study. The swab samples and extracted DNA samples were carefully identified and kept in separate boxes.

*Statistical analysis* - Data were entered in a spreadsheet using Microsoft Excel 2011 for Mac (Microsoft Corp, USA) and transferred to SPSS 16.0 statistical software (SPSS Inc, USA). A bivariate analysis for all variables of interest was performed for the case and control data. The chi-square test and Fisher's exact two-tailed test analysis was performed and differences were considered significant at values of p < 0.05. Stata v.12.0 (Stata Corp, USA) was also used to further analyse the data. Variables with p value < 0.20 were included into a logistic regression analysis in order to investigate if RLEP PCR positivity can predict cases and controls adjusting for other studied variables. Variables with p value < 0.05 were kept in the final model (age, sex, SES and history of a previous contact with a leprosy case). For logistic regression the forward stepwise method and Wald statistic were used. PCR was kept in the model as an independent variable to emphasise that it is not related to C and Co.

*Spatial analysis* - We conducted a spatial analysis of RLEP positive prevalence in nasal secretion in the three groups (C, Co and EG). All participants were geolocated using Google Earth software and a database of street network files. The coordinates were transcribed to the Excel spreadsheet and subsequently transformed into PDF files to be used directly in the Geographic Information System application ArcGIS 9.3 (Environmental Systems Research Institute, USA). After the data had been formatted in spreadsheets, ArcMap was used to illustrate the spatial distribution of cases and controls in Fortaleza. Population densities were generated using the Spatial Analyst extension - kernel density, using a 20 km search radius and a grid size of 2.5 km.

*Ethics* - All participants signed an informed consent form and authorised the collection of samples. This project was approved by the CDERM Ethical Committee (protocol 011/07) and guidelines of the Ethical Committee were followed in conducting the research.

## RESULTS

Among the 185 leprosy cases, 89 were of borderline clinical form, 37 were tuberculoid, 44 were lepromatous and 9 were indeterminate. Six cases were not classified according to their clinical form. Men predominated among C (p < 0.0001) (data not shown). The age of cases ranged from 4-81 years, similar to Co (4-69 years old), but the overall mean age of the C (40.3 years) was higher than the Co (29.1 years; p < 0.0001). In the amplification analysis of RLEP with PCR, 69.2% of cases were positive, with a similar result seen among Co (66.9%; p = 0.7163) (Table I). For the EG ages (data not shown) ranged between 18-31 years (61.2% females and 38.8% males) and the prevalence of RLEP positive among the EG was 28.1%.

**TABLE I:** Risk factors related to leprosy cases (C) and controls (Co)

Variables	C (n = 185) n (%)	Co (n = 136) n (%)	p^*a*^	OR (95% CI)
Sex				
Male	99 (53.5)	33 (24.3)	< 0.0001	3.593 (2.208, 5.847 )
Female	86 (46.5)	103 (75.7)	-	1.000
Age (years)				
Mean (range)	40.3 (4-81)	29.1 (4-69)	< 0.0001^*b*^	1.045 (1.029, 1.061 )
*Mycobacterium leprae* RLEP				
PCR positive 219 (68.2%)	128 (69.2)	91 (66.9)	0.7163	1.110 (0.691, 1.784)
PCR negative 102 (31.8%)	57 (30.8)	45 (33.1)	-	1.000
Education (years)				
< 4	110 (59.5)	56 (41.2)	< 0.0001	3.036 (1.763, 5.227 )
4-8	42 (22.7)	29 (21.3)	-	2.238 (1.175, 4.265 )
> 8	33 (17.8)	51 (37.5)	-	1.000
Education of the head of the family (years)				
< 8	118 (69.0)	57 (43.2)	< 0.0001	2.929 (1.826, 4.701 )
> 8	53 (31)	75 (56.8)	-	1.000
Socioeconomic status				
B/C	61 (33)	83 (61)	< 0.0001	1.000
D/E	124 (67)	53 (39)	-	3.183 (2.007, 5.049 )
BCG scar				
Yes	115 (63.9)	105 (79.5)	0.0037	1.000
No	65 (36.1)	27 (20.5)	-	2.198 (1.306, 3.701 )
History of a previous contact with a leprosy case				
Yes	114 (61.6)	46 (33.8)	< 0.0001	3.141 (1.978, 4.989 )
No	71 (38.4)	90 (66.2)	-	1.000

*a*: Fisher exact test; *b*: Mann Whitney *U* test; CI: confidence interval; OR: odds ratio; PCR: polymerase chain reaction; RLEP: *M. leprae*-specific repetitive element.

Educational level, SES and presence of Bacillus Calmette-Guérin (BCG) scar were included in the study. Educational level is traditionally used as an indicator of SES in Brazil. Individuals with a lower educational level (< 4 years of schooling) had a higher risk of developing leprosy [59.5% vs. 41.2%; p < 0.0001, odds ratios (OR) = 3.036]. A similar trend was found for cases with low incomes; SES D/E was more common among leprosy cases compared to the Co individuals (67% vs. 39%; p < 0.0001, OR = 3.183). In addition, a higher risk for developing leprosy was found for those whose head of the family had a lower level of education (69% vs. 43.2%; p < 0.0001, OR = 2.929). Among the leprosy cases, the absence of BCG scar was significantly higher compared to the Co group (36.1% vs. 20.5%; p = 0.0037, OR = 2.198). History of a previous contact with a leprosy case was reported to 61.6% and 33.8% of the C and Co, respectively (p < 0.0001, OR = 3.141) (Table I).

Positivity by clinical classification is presented in Table II. We observed no difference in PCR positivity related to the different clinical forms. However and as expected, we observed a strong positive correlation between DNA detection in nasal swabs for the leprosy cases and the BI (78.8%, p = 0.026).

**TABLE II:** Leprosy cases by bacilloscopy index and Ridley-Jopling clinical classification

Variables	PCR+ n (%)	PCR- n (%)	p^*a*^	OR (95% CI)
Bacilloscopy index				
Positive	67 (78.8)	18 (21.2)	0.0260	2.233 (1.121, 4.451)
Negative	50 (62.5)	30 (37.5)	-	1
Clinical classification				
Borderline	58 (65.2)	31 (34.8)	0.3746	-
Tuberculoid	27 (73)	10 (27)	-	-
Lepromatous	34 (77.3)	10 (22.3)	-	-
Indeterminate	5 (55.6)	4 (44.4)	-	-

*a*: Fisher exact test; CI: confidence interval; OR: odds ratio; PCR: polymerase chain reaction.

A multivariate logistic regression examined the risk factors associated to leprosy cases and controls adjusting for all the other variables with p < 0.20, including being positive to *M. leprae* DNA in nasal secretion. Being older {OR = 1.048 [(95% confidence interval (CI) 1.029-1.068]}, male [OR = 6.240 (95% CI 3.356-11.601)], belonging to a lower (D/E) socioeconomic class [OR = 3.347 (95% CI 1.906-5.879)] and reporting a history of previous contact with a leprosy case [OR = 3.859 (95% CI 2.206-6.752)] were more likely to be a leprosy case. Contrary to our hypothesis, being RLEP PCR positive was not found to be related to cases or controls [OR = 1.062 (95% CI 0.594-1.897)] (Table III).

**TABLE III:** Logistic regression*a* to access risk factors associated with leprosy cases (C) and controls (Co)

Factors	p^*a*^	Cases
		OR (95% CI)
Age (years)^*b*^	< 0.001	1.048 (1.029, 1.068)
Male sex^*b*^	< 0.001	6.240 (3.356, 11.601)
Socioeconomic status (D/E class^*b*^)	< 0.001	3.347 (1.906, 5.879)
History of a previous contact with a case^*b*^	< 0.001	3.859 (2.206, 6.752)
PCR^*c*^	0.594	1.062 (0.594, 1.897)

*a*: Wald statistic; *b*: forward stepwise method adjusted to all variables in the model;*c*: polymerase chain reaction (PCR) was kept in the model to show it is not related to C and Co; OR: odds ratio; CI: confidence interval.

**Fig. 1:**
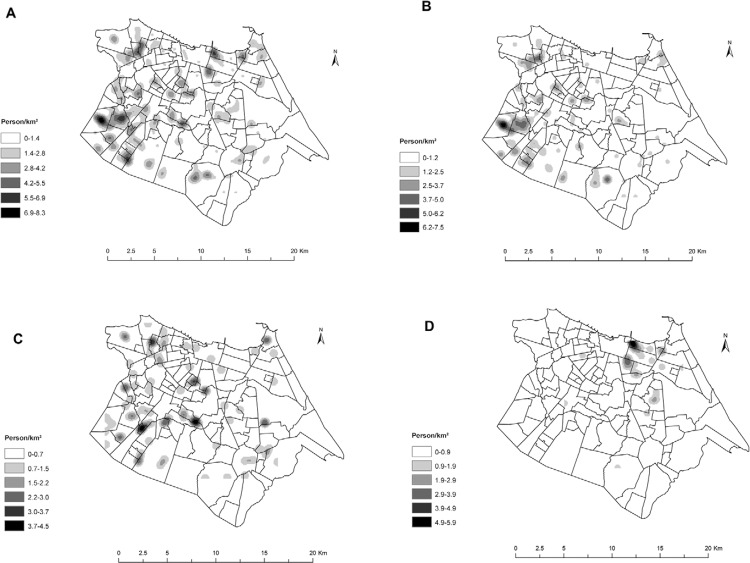
spatial kernel density of specific repetitive element (RLEP) polymerase chain reaction (PCR) positive of *Mycobacterium leprae* DNA from nasal samples. A: All RLEP PCR positivity studied individuals [cases (C), controls (Co) and external groups (EG)]; B: C; C: Co; D: EG.

**Fig. 2:**
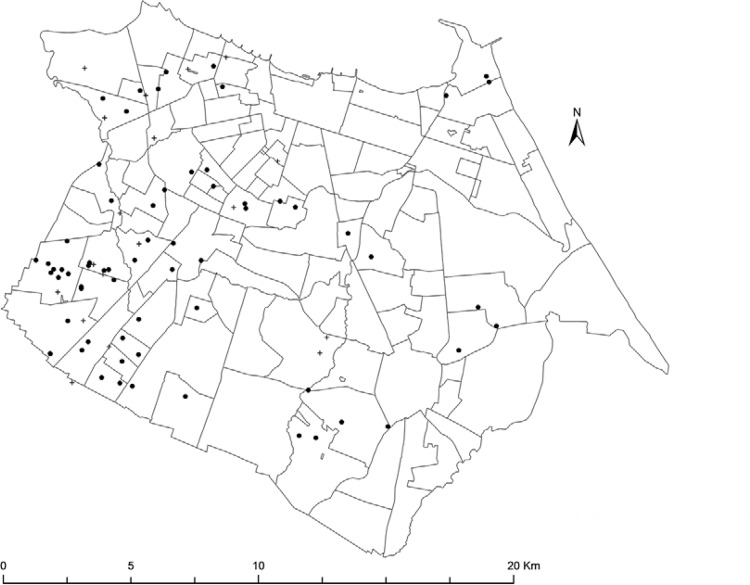
map of the city of Fortaleza, state of Ceará, Brazil, showing leprosy cases with bacilloscopy index positive and specific repetitive element (RLEP) polymerase chain reaction (PCR) from nasal samples. ●: leprosy cases with positive RLEP PCR; +: leprosy cases with negative RLEP PCR.

The geographical kernel density distribution of all members with *M. leprae* DNA positive in nasal secretion in Fortaleza is illustrated inFig. 1A. We observed that the groups C and Co were concentrated in the southwest and western side of Fortaleza (Fig. 1B, C), areas of lower SES, while group EG is concentrated in clusters in the northeast region of the map (Fig. 1D), representing wealthier areas of the city. In addition, cases with a positive bacilloscopy and RLEP positive are also clustered (Fig. 2). The distance between the cases that comprised each cluster varied from 121-1,000 m.

## DISCUSSION

This study found a very high PCR positivity of RLEP DNA in nasal samples for both leprosy cases and their controls. These results are much higher than in endemic areas of Indonesia, 6.6% (Bakker et al. 2006) and 7.8% (Klatser et al. 1993). However, results are similar to studies conducted in the Philippines (55%) (de Wit et al. 1993) and Brazil (58.3%) (Patrocinio et al. 2005).

Our study showed that PCR positivity for controls was very similar to that of cases. Both populations inhabit the poorest part of Fortaleza, but controls are geolocated in bridging areas that connect the richest part of the city with the poorest area. CDERM is a public dermatology centre drawing mainly low-income patients that live in poorer areas of Fortaleza. These areas are hyperendemic for leprosy, present the lowest family income and the worst general health indicators in the city (ABEP 2014, IPECE 2014, SESA 2014). Individuals, including both C and Co, work throughout the city and many of the poorest work as domestics and food handlers in wealthy areas of the town. Additionally, while Co live in the same areas as C, the spatial analysis shows that they are more spread out geographically and sometimes overlap in areas where higher social status EG live. Thus, active bacilli transmission between these two groups might occur in several settings. Detection of the bacilli may be demonstrated in studies among contacts showing rates of positivity ranging from 1.7-23% (de Almeida et al. 2004, Nagao-Dias et al. 2007, Banerjee et al. 2010, Custodio et al. 2012).

Since the primary site of infection with *M. leprae* as a result of airborne infection is *via* the nose, PCR assays have been used to detect leprosy in nasal secretions in endemic communities. While a positive PCR from nasal mucosa do not differentiate a contact from a case in an endemic area, it does offer the opportunity to follow-up PCR positive individuals to identify new cases. Interestingly, Martins et al. (2010) report that many people discovered with PCR positive nasal secretions also report a nonspecific rhinopathy.

This study showed the bacilli are found in cases and controls with no statistical difference between the two groups. This means that individuals only will develop the disease if there is an association with other risk factors, including socioeconomic and demographic conditions, previous contact with a leprosy case and BCG status.

Our data show a statistical age difference among cases and controls. At the same time there was no difference between the proportions of leprosy carriage among the two groups. Is important to note that for each year of age the chance to become a case increases exponentially 5% per year, after adjusting for all the other studied variables. Age has been previously found to be a risk factor for leprosy in CE (Frota et al. 2010). Leprosy has a long incubation period - usually two-seven years - and therefore cases tend to be older than healthy individuals (Kerr-Pontes et al. 2004). In an endemic region, the age difference reflects the time that an individual can be exposed to the bacillus and the risk to later develop the disease.

The EG group showed the lowest rate of positives. The different detection rates found in groups Co and EG are associated with differences in the SES of the members of the groups. While participants of Co are mostly people with low SES and living in poorer areas of the city, EG are members of high-income families (Fig. 1). Despite the lower PCR positivity in EG, levels reported are higher than published previously (de Almeida et al. 2004, Nagao-Dias et al. 2007, Banerjee et al. 2010, Custodio et al. 2012). Moreover, studies indicate that contacts of leprosy cases that are nasal PCR positive have a higher risk of developing the disease (Douglas et al. 2004, Bakker et al. 2006).

Since Fortaleza is a leprosy endemic area, we postulate that Co acquires the bacilli from C and becomes a healthy nasal carrier that silently, but actively transmits the*M. leprae *to the EG individuals. In agreement with our findings, previous studies have shown that individuals may be exposed to a source of infection from carriers who harbour bacilli in their nose even for a short period of time (Patrocinio et al. 2005, Araújo et al. 2012). As seen in Fortaleza and other places, PB cases probably account for a significant part of the transmission in endemic communities (Beyene et al. 2003,Martins et al. 2010).

In the geographic distribution analysis of PCR positivity among the cases with a positive bacilloscopy index in Fortaleza, individuals were found clustered in certain small areas. As expected, the areas surrounding those clusters were found to have a significantly higher concentration of leprosy cases. These data suggest that MB cases harbouring *M. leprae *in nasal cavities could be a major potential pathway for transmission of the bacillus within the population.

Among RLEP positives, we found men more likely to be cases and women to be controls (57.8% vs. 78%, OR = 4.865; 95% CI, 2.650-8.932; data not shown). In the context of multivariate analysis, we found men 6.24 more likely to be a case than women, but PCR was not found to be related to being a case. In a cohort study (Bakker et al. 2006) conducted in the Flores Sea Islands of Indonesia, it was shown that males have twice the risk of acquiring leprosy compared to women. A previous study using anti-phenolic glycolipid (PGL)1 conducted by our group in other endemic areas of CE, also found a higher proportion of seropositivity among men. In a separate paper in preparation using social network analysis we hypothesise that men have contact occurring at the work place.

The highest rate of PCR positivity was among the cases with positive bacilloscopy, as these individuals have a high bacillary load (high BI). However, there was no difference in PCR positivity among the different clinical forms of the disease. Other studies have found a higher PCR positivity in nasal secretion among the MB clinical form compared to the PB ones (Banerjee et al. 2010, Araújo et al. 2012). We hypothesise that *M. le- prae* is so widespread across the city that patients harbour not only their own bacilli, but also bacilli from multiple infections from other patients/carriers that they are exposed to. This may be one reason we cannot differentiate nasal carriage by clinical form.

The BCG vaccine - originally used to protect against tuberculosis - has been shown to provide protection against leprosy. Most vaccinated seropositive contacts who develop leprosy display the mildest form of the disease (PB) with negative serology for *M. leprae *(Rodrigues et al. 2007, Lobato et al. 2011). A negative association was found between subjects with BCG scar and cases. These results corroborate other studies that demonstrate the protection of BCG in leprosy prevention (Kerr-Pontes et al. 2006, Zodpey 2007, Merle et al. 2010). BCG improves the immunological response of leprosy cases by inducing the activation of the initial phase of immunocellular activity, innate human immunity (increase in tumour necrosis factor alpha, interleukin-12 and macrophage activation) (Zenha et al. 2012).

Several studies show a correlation between higher incidences of leprosy and lower SES (Kerr-Pontes et al. 2006, Sales et al. 2011, Cury et al. 2012). In agreement with the literature, individuals belonging to socioeconomic class D/E were found to be 3.35 times more at risk to be a leprosy case than controls. Lower SES is strongly related to low educational achievement. In this study illiteracy and educational attainment of less than eight years of schooling for the head of household was associated with an increased chance of being a leprosy case. Low educational level has been reported as a risk factor for leprosy in studies conducted in CE (Kerr-Pontes et al. 2004, 2006).

PCR can be used to complement leprosy diagnosis and classification and can play a role in choosing the most appropriate treatment, thereby reducing the risk of disability and disease transmission. Using PCR, this study showed a high rate of positivity among the three groups, demonstrating many individuals colonised with*M. leprae* in the upper airways that may be taking an active role in the transmission of leprosy. This study used the RLEP repetitive element sequence of *M. leprae*, which is reported to be specific for*M. leprae* and is not present in other mycobacterial or bacterial species (Wen et al. 2013). The RLEP-based PCR is capable of detecting *M. leprae* DNA in 73% of patients with a BI of 0 (Woods & Cole 1989, Yoon et al. 1993). In addition, a nested PCR based on the RLEP region was shown to amplify 1 fg of purified DNA and was used to detect DNA in patients' whole blood, with higher sensitivity and specificity (Wen et al. 2013). All samples tested for RLEP sequence were also submitted to amplification with *rpoT* primers demonstrating an agreement in the results (Supplementary Figs 1-3). Since we used several strategies to minimise false-positive amplifications and contamination, we are confident that these are valid data. Corroborating these findings, the anti-PGL-I seropositivity of contacts of leprosy cases were much higher than reported in our previous study in a hyperendemic region of CE (Frota et al. 2010).

Even though our study demonstrated that C and Co did not differ in the positivity of RLEP PCR in nasal samples we encourage testing of nasal swabs among cases, contacts and noncontacts of cases in areas where cases live, since positive noncases are more likely to turn into a case. The World Health Organization proposes clinical diagnosis as a main strategy to identify cases. Given the difficulties of clinical diagnosis and the failure of this current strategy, at least in Brazil, the molecular techniques we report here show real promise. Our results would argue that collecting and testing nasal swabs could be an important strategy for identifying cases and carriers in the population living in hyperendemic clusters where cases are concentrated. Although nasal carriage does not necessarily imply infection or excretion of bacilli, our findings support the hypothesis that in endemic areas like Fortaleza, *M. leprae* is disseminated among the general population. As molecular epidemiology techniques become cheaper these active surveillance procedures might finally achieve the goals of leprosy control promised by the advent of therapies more than 50 years ago.
