# Supplementary material for: Widespread nasal carriage of Mycobacterium leprae among a healthy population in a hyperendemic region of northeastern Brazil
Source: Mem Inst Oswaldo Cruz. 2015 Nov;110(7):898–905. doi: 10.1590/0074-02760150178 (PMC4660619; doi:10.1590/0074-02760150178)
Supplement: Supplementary file 1 [file 0074-0276-mioc-110-7-0898-suppl.pdf]

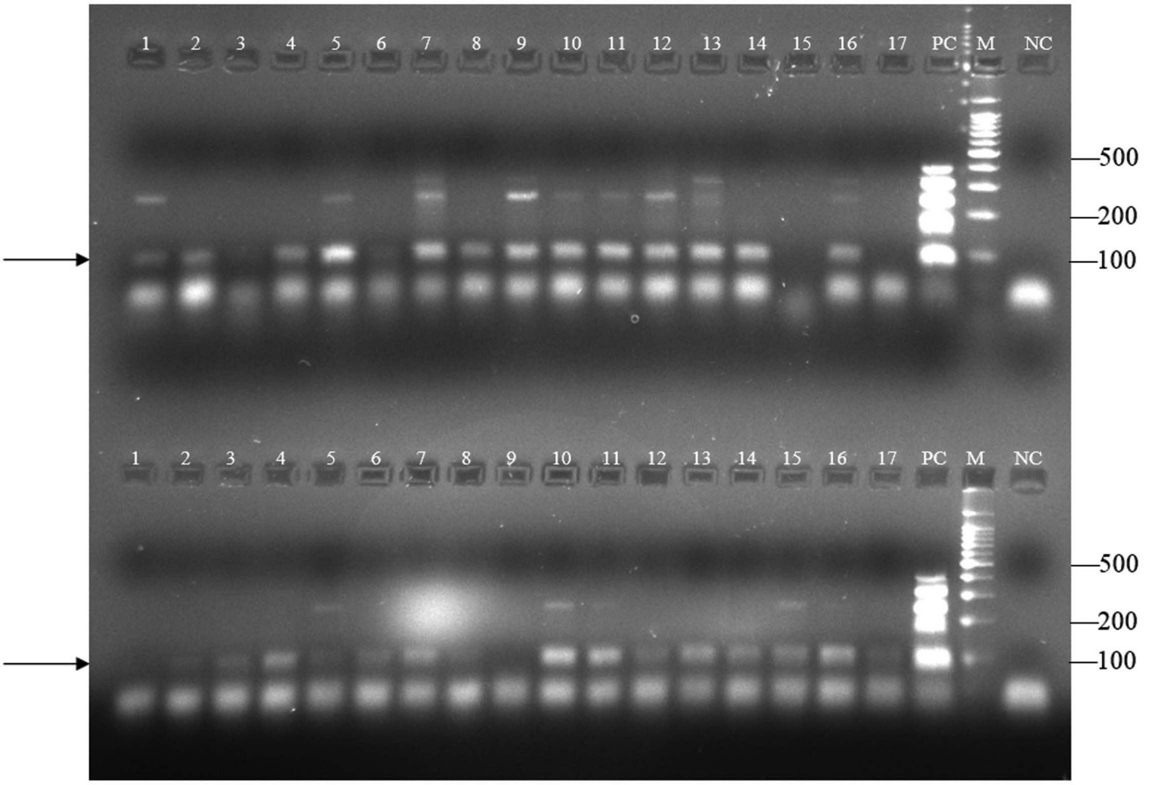

Fig. 1: agarose gel analysis of 91 bp DNA product (black arrows) for the *rpoT* region of *Mycobacterium leprae* by variable number tandem repeat (combination 1) polymerase chain reaction in nasal samples from healthy controls (Co). Upper panel Lanes 1, 2, 4-14, 16: amplified DNA Co samples; 3, 15, 17: nonamplified Co; lower panel 3-7, 10-17: amplified DNA Co samples; 1, 2, 8, 9: nonamplified Co samples; M: 100 bp DNA ladder; NC: negative control (distilled water); PC: positive control (20 pg of chromosomal *M. leprae* DNA).

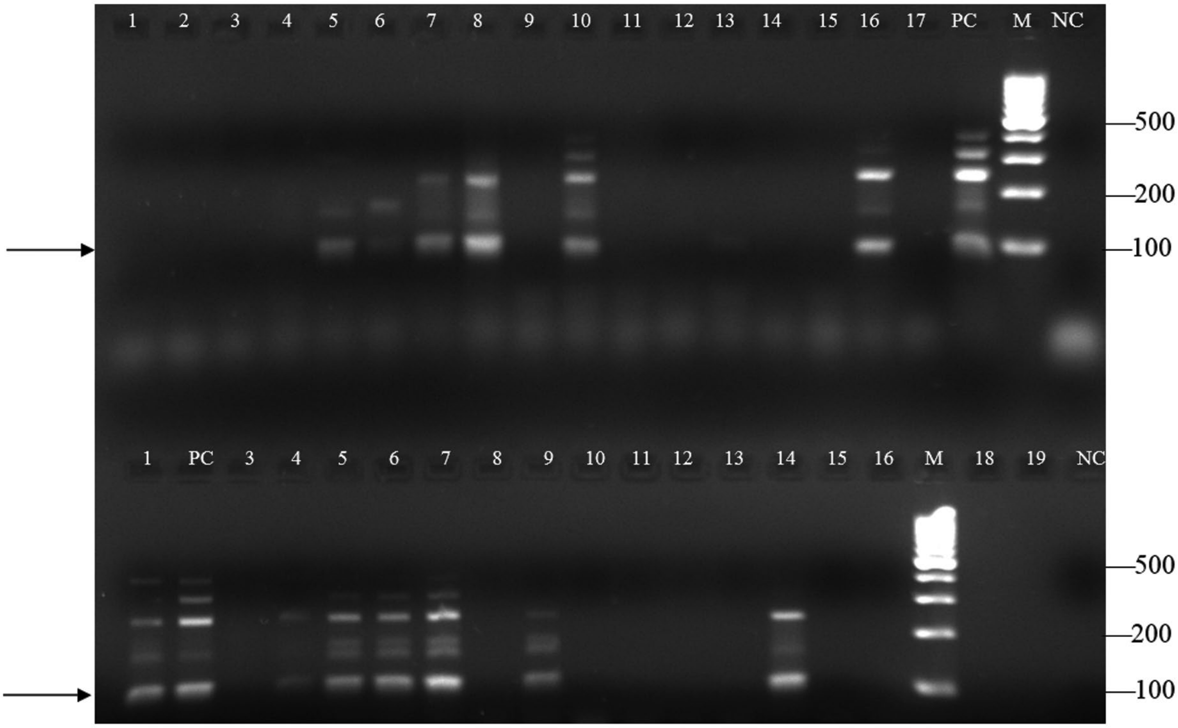

Fig. 2: agarose gel analysis of 91 bp DNA product (black arrows) for the *rpoT* region of *Mycobacterium leprae* by variable number tandem repeat polymerase chain reaction in nasal samples from leprosy cases (C) and healthy controls (Co). Upper panel Lanes 5-8, 10, 16: amplified DNA of Co samples; 1-4, 9, 11-15, 17: nonamplified Co samples; lower panel 1, 4-7, 9, 14: amplified DNA of C samples; 3, 8, 10-13, 15-16: nonamplified C samples; 18, 19: empty; M: 100 bp DNA ladder; NC: negative control (distilled water); PC: positive control (20 pg of chromosomal *M. leprae* DNA).

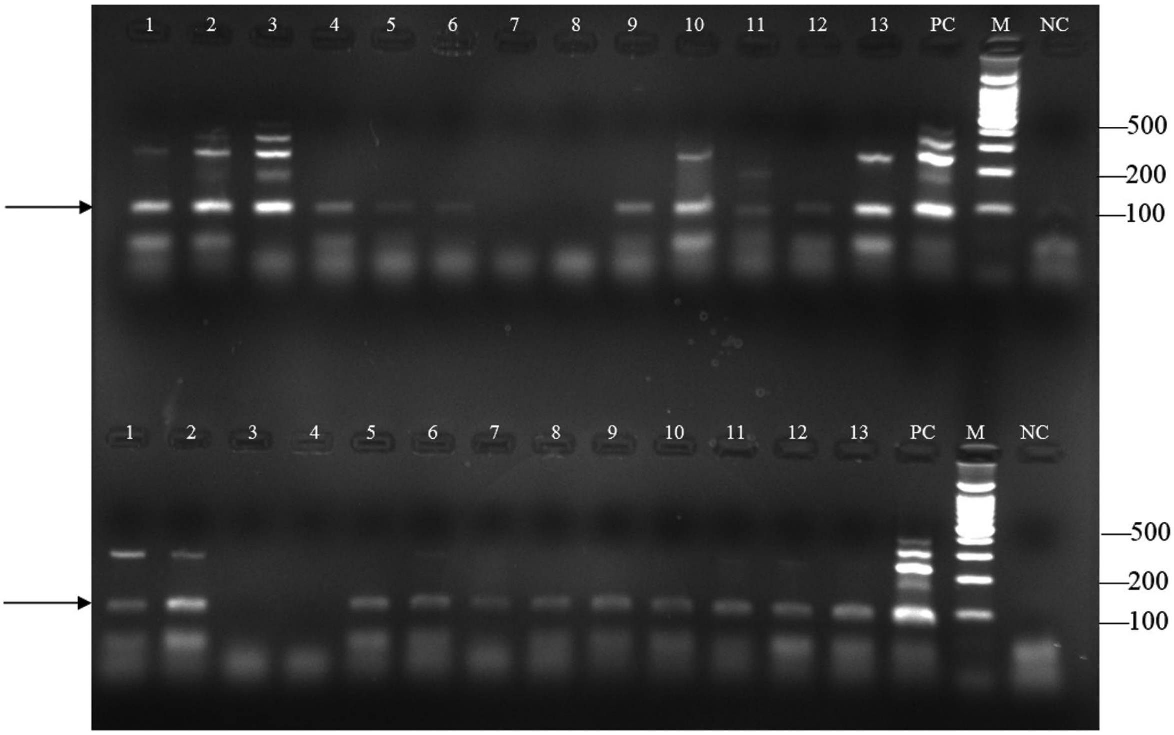

Fig. 3: agarose gel analysis of 91 bp DNA product (black arrows) for the *rpoT* region *Mycobacterium leprae* by variable number tandem repeat (combination 1) polymerase chain reaction in nasal samples from leprosy cases (C). Upper panel Lanes 1-6, 9-13: amplified DNA C samples; 7, 8: nonamplified C samples; lower panel 1, 2, 5-13: amplified DNA C samples; 3, 4: nonamplified C samples; M: 100 bp DNA ladder; NC: negative control (distilled water); PC: positive control (20 pg of chromosomal *M. leprae* DNA).
